# Sensor-Based Classification of Primary and Secondary Car Driver Activities Using Convolutional Neural Networks

**DOI:** 10.3390/s23125551

**Published:** 2023-06-13

**Authors:** Rafał Doniec, Justyna Konior, Szymon Sieciński, Artur Piet, Muhammad Tausif Irshad, Natalia Piaseczna, Md Abid Hasan, Frédéric Li, Muhammad Adeel Nisar, Marcin Grzegorzek

**Affiliations:** 1 Department of Biosensors and Processing of Biomedical Signals, Faculty of Biomedical Engineering, Silesian University of Technology, Roosevelta 40, 41-800 Zabrze, Poland; szymon.siecinski@polsl.pl (S.S.); natalia.piaseczna@polsl.pl (N.P.); 2 Institute of Medical Informatics, University of Lübeck, Ratzeburger Allee 160, 23562 Lübeck, Germany; ar.piet@uni-luebeck.de (A.P.); m.irshad@uni-luebeck.de (M.T.I.); md.hasan@student.uni-luebeck.de (M.A.H.); fr.li@uni-luebeck.de (F.L.); marcin.grzegorzek@uni-luebeck.de (M.G.); 3 Department of Information Technology, University of the Punjab, Lahore 54000, Pakistan; adeel.nisar@pucit.edu.pk; 4 Department of Knowledge Engineering, University of Economics in Katowice, Bogucicka 3, 40-287 Katowice, Poland

**Keywords:** driving a car, driving behavior, electrooculography, convolutional neural networks

## Abstract

To drive safely, the driver must be aware of the surroundings, pay attention to the road traffic, and be ready to adapt to new circumstances. Most studies on driving safety focus on detecting anomalies in driver behavior and monitoring cognitive capabilities in drivers. In our study, we proposed a classifier for basic activities in driving a car, based on a similar approach that could be applied to the recognition of basic activities in daily life, that is, using electrooculographic (EOG) signals and a one-dimensional convolutional neural network (1D CNN). Our classifier achieved an accuracy of 80% for the 16 primary and secondary activities. The accuracy related to activities in driving, including crossroad, parking, roundabout, and secondary activities, was 97.9%, 96.8%, 97.4%, and 99.5%, respectively. The F1 score for secondary driving actions (0.99) was higher than for primary driving activities (0.93–0.94). Furthermore, using the same algorithm, it was possible to distinguish four activities related to activities of daily life that were secondary activities when driving a car.

## 1. Introduction

To drive safely, the driver must be sufficiently aware of his/her surroundings, pay constant attention to the road and traffic, and be alert enough to react to unexpected circumstances [1,2,3,4]. Tasks that are directly related to maneuvering a vehicle are called basic driving activities [5].

The lack of concentration of drivers remains one of the crucial factors that contribute to serious accidents and deaths on the road and continues to be a problem for international road safety measures, as they affect not only the driver but also everyone else on the road [6,7]. Approximately 324,000 people were injured due to driver inattention in the United States in 2020 and more than 3000 lost their lives, representing 8.1% of all fatal accidents in the country [8,9].

The term “driver fatigue” refers to a particular type of inattention that occurs when a driver removes his/her focus from basic vehicle navigation tasks to focus on another activity [10]. These distractions may come from common activities, such as talking to other passengers and eating, as well as using mobile phones and systems [11]. These activities can have different effects on drivers. From the point of view of support by measurement technologies, existing research indicates two main areas:

1. Detect anomalies in driver behavior to prevent an accident, with personalized behavior measures of driving style through face detection or Internet of Things (IoT) technologies [12,13,14,15,16];
2. Monitor correct cognitive and safe driver behaviors with intelligent sensors and IoT to monitor the face, eyes, or movements of a driver’s entire body for a novel driver education process [17].

Although there are two distinct categories, they have a lot in common; for example, they are recorded and classified within the framework of currently available technologies, and many activities can be assigned into more than one of them. The use of a device requires participation in all of these distractions, also known as secondary driving activities. The cognitive distraction that occurs in the driver’s brain is the most difficult to identify. This phenomenon is also known as “looking but not seeing”. Attention requirements for distracting work and the prevalence of multitasking among drivers are two fundamental elements of the problem of distributed driving safety [18,19,20].

Task demand is the total amount of visual, physical, and cognitive resources required to perform the activity. The second issue is the frequency with which the drivers perform the task. Even a task that is small, but performed frequently, can pose a safety concern [2,3,19,21,22].

According to [23,24], the results suggest that activities that require the driver to look away from the road or perform manual tasks significantly increase the probability of a collision. The risk of a traffic accident increases by 2.05 when using a mobile phone, especially when dialing (×12) and sending messages (×6).

The long time spent looking away from the road also has a significant impact. According to some studies, removing your eyes from the road for more than two seconds significantly increases the probability of safety-critical events [25]. In fact, the U.S. Department of Transportation advises against taking your eyes off the road repeatedly in 12 s while operating a motor vehicle [26]. Recognition of human activity based on preconstructed groups of activities is a commonly used approach [27,28,29,30].

There are many well-described activities, mainly related to basic needs and daily life, e.g., breathing, eating, sleeping, and walking [31,32,33,34,35]. Among the recognition of these activities, some were divided into even more detailed (complex) activities, e.g., food was divided into food preparation, and food preparation was even more separated for the preparation of breakfast, lunch, and dinner. Using this convention, we decided to analyze and recognize the activities and scenarios that accompany driving a vehicle.

To explore more deeply the research problem, our objective was two-fold: to identify the prevailing road conditions during a trip and to determine whether individuals exhibited improved parking skills after the journey. Accomplishing this required the acquisition of a substantial volume of data. This paper outlines the integration of data and fundamental principles of physics into sensors embedded within JINS MEME ES_R glasses, as well as the methodology employed to acquire and analyze the collected data for classification purposes [3,36,37,38,39,40].

To summarize, we make the following contributions:

1. We investigate the use of JINS MEME ES_R (smart glasses) sensor data and develop a state-of-the-art machine learning model that learns patterns related to the primary and secondary activities of drivers and classifies them into their respective classes.
2. We perform a comparative analysis of wearable sensor data consisting of nine activities of the first driver and four activities of the second driver.
3. We provide a brief review of related approaches.

The rest of the article is structured as follows: Section 2 presents the current state of the art in the field of recognition of vehicle driver activities. Section 3 describes the materials and methods used to analyze signals to assess these activities. Section 4 presents the experimental results. Section 5 provides a discussion and, finally, Section 6 concludes this work.

## 2. Related Works

When looking for examples of similar studies to compare, it should be noted that, in a ratio of four to one, articles were found dedicated to searching for anomalies such as drowsiness, fatigue, lack of driver concentration, and external factors associated with vehicle damage and atmospheric factors associated with driving conditions [41,42,43].

In another study based on data tracking the head and eyes in driving simulation conditions, the activity of 73 people who performed various secondary tasks while driving was recorded. The results of this research improved performance classification through the development of new functions, in particular to assess the context of autonomous driving [44]. Algorithms for the classification of eye movements were divided into methods based on statistical thresholds and probabilistic methods. Algorithms based on static thresholds are usually selected for the classification of tasks assigned to the person who performs them; in other words, they are limited in quantity.

Probabilistic methods were introduced to meet the challenge of automatic adaptation of many people as a result of various behaviors, for example, individual visual cues. Drowsiness while driving is a critical issue in the context of road safety. Several approaches have been developed to reduce the risk of driver drowsiness. Fatigue and drowsiness detection techniques are divided into three broad strategies, namely vehicle-based, physiological, and driver-based approaches. This article discusses the latest research on diagnosing driver drowsiness based on behavior, in particular changes in eye movements and facial features.

### 2.1. Drowsiness in Drivers

Another research project turned to a traffic surveillance system developed to detect and warn the driver of a degree of drowsiness or stress [45,46,47]. A smartphone with a mobile application, using the Android operating system, was used to implement a human–computer interaction system. To detect drowsiness, the most important visual indicators that reflect the driver’s condition are the behavior of the eyes, the side and front of the head, and yawning. The system works well under natural light conditions and regardless of the use of accessories supplied by the driver, such as glasses, hearing aids, or a cap.

Due to the large number of road accidents in which drivers fall asleep, this project was implemented to develop methods to prevent napping by providing a non-invasive system that is easy to operate and without the need to purchase additional specialized equipment. This method was able to detect drowsiness with an efficiency of 93.4% [48].

Another significant educational research experiment evaluated how an educational program affected the fatigue and conduct of teenage and adult drivers, as well as their performance and behavior during simulated driving at night. A 4-week sleep program and a 4-week driving program were randomly assigned to 34 volunteers (aged 18 to 26). The findings imply that the educational program increases people’s awareness of sleepiness. Sleep and driving instruction can reduce the risk that young drivers become fatigued and suffer accidents related to fatigue, but this requires a more comprehensive evaluation of their real driving abilities [49].

### 2.2. Wireless Sensor Networks

Next, we consider a second group of studies related to eliminating typical driver behavior and IoT-based traffic management to increase road safety. IoT is an innovative design paradigm designed as a network of billions to trillions of tiny sensors communicating with each other to offer innovative solutions to problems in real time [50]. These sensors form a network called a wireless sensor network (WSN) to monitor the physical environment and distribute the collected data back to the base station via multiple hops.

WSN has the ability to collect and report data for a specific application. Location information plays an important role in various wireless sensor network applications. Therefore, such systems can improve driving safety. However, real-time monitoring of driving behavior and conditions is linked to various issues, including dizziness caused by long journeys, drastic changes in lighting, and reflections in a driver’s glasses.

### 2.3. Deep Learning and Driver’s Gaze

A deep learning approach was presented in [51,52,53,54] to address this problem, where the authors used a near-infrared (NIR) camera sensor to detect glances, as well as head and eye movements, without the need for user calibration at first. The proposed system was evaluated on a dedicated database, as well as on Columbia’s open dataset (The Face Tracer CAVE-DB database).

A comprehensive solution was introduced in previous works [51,52,53,54] to address the aforementioned issue by employing deep learning models. This approach used a near-infrared (NIR) camera sensor to accurately identify glances, head movements, and eye movements, all without the need for initial user calibration. The efficacy of the proposed system was assessed on a specialized database and additionally validated using Columbia University’s publicly accessible dataset, known as The Face Tracer CAVE-DB database.

The driver’s gaze turned out to be an excellent way to create a system for driving intelligent vehicles. Due to the fashion for highly autonomous vehicles, the driver’s view can be useful in determining the time of transmission of the gesture from the driver to the traffic management system. Although there have been significant improvements in the personalization of driver vision assessment systems, a universal generalized system that is immutable for different perspectives and scales has not yet been developed. We are taking a step towards this general system using convolutional neural networks (CNNs).

The utilization of the driver’s gaze has emerged as a promising avenue for developing intelligent driving systems. In the context of the rising popularity of highly autonomous vehicles, leveraging the driver’s perspective becomes crucial in accurately timing the transmission of gestures to the traffic management system. Despite notable advances in tailoring driver vision assessment systems to individual users, a universally applicable and adaptable system, capable of accommodating diverse perspectives and scales, remains an open problem. To address this challenge, we are progressing towards the development of a comprehensive framework using convolutional neural networks (CNNs), aiming to establish a generalized solution.

In [55,56] four prominent convolutional neural network (CNN) architectures specifically designed for this purpose were used to conduct detailed comparisons of their performance. Additionally, various modifications were applied to the input images and the influence of the image size on the effectiveness of the models was examined.

To facilitate network training and evaluation, a substantial dataset was collected comprising 11 extended driving activity recordings. This dataset encompassed the driving behaviors of 10 individuals in two distinct vehicles. The most successful models achieved a recognition accuracy of 95.2% during the comparative testing phase.

Subsequently, the highest performing model was subjected to a comparison with the publicly available Columbia Gaze dataset. This dataset consisted of images showing 56 individuals displaying various head positions and viewing directions. Interestingly, even without any specific training on this particular dataset, the model effectively interpreted different perspectives from disparate datasets [57].

## 3. Materials and Methods

**Figure 1:**
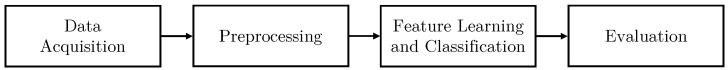
Standard approach to developing a deep learning model. Each step in the chain should be optimized in parallel to achieve the best possible performance.

This section presents details on the sensor modalities that were used for data acquisition, discusses the data acquisition process, and explains the experimental settings. Figure 1 shows all the steps in the process from data acquisition to evaluation, which has been extensively described in [40,58,59].

### 3.1. Data Acquisition

We acquired the dataset using JINS MEME smart glasses, which have a six-axis inertial measurement unit (IMU) that incorporates EOG, an accelerometer and a gyroscope [60,61]. Participants volunteered for the study and gave their informed consent.

**Figure 2:**
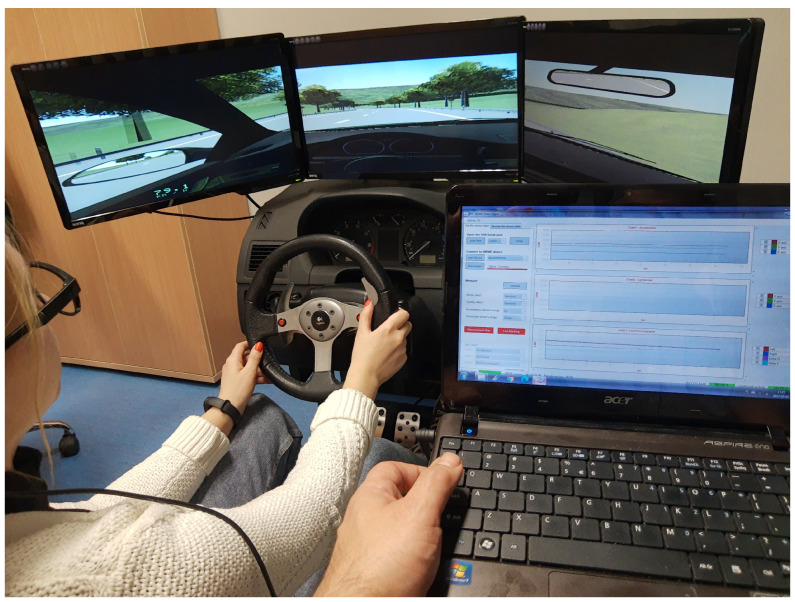
Driving simulator setup used for the data acquisition.

The experiments were carried out in a simulated environment [11,62] as presented in Figure 2.

The simulator consists of the following components:

- A central unit equipped with:
  – An Intel Core i7 processor;
  – XFX RADEON HD 5770 1 GB graphic card with NVIDIA processor and 3D VISION system;
  – 4 GB memory;
  – Gigabyte’s Ultra Durable 3 motherboard;
- A special construction made of steel;
- A two-way adjustable seat;
- A Logitech set: steering wheel, pedals, and gearbox;
- Three LED 27 monitors suitable for long operation;
- A sound system;
- Dedicated software “Nauka jazdy” (English: Driver training).

The study consisted of two independent experiments that were conducted separately. Both were completed using the JINS MEME ES_R software with the default settings. The EOG sampling rate was 200 Hz, the accelerometer sampling frequency was 100 Hz, and the accelerometer measurement range was ±2 g. We synchronized all frequencies to 50 Hz. The signals were recorded simultaneously for each subject while they received voice commands during the driving simulation.

Nine subjects in total (five men and four women) volunteered to participate in the study. Six individuals, all graduate students in their 20s, four men and two women, performed the fundamental driving tasks. In total, we collected 1200 samples of primary driver activities, evenly divided into classes that represent a different activity. Half of the samples were created by one participant, while the remaining samples were evenly distributed among the other subjects.

For the secondary driver activities, we recorded 700 samples that were distributed equally among all classes. Four subjects, one male and three female, with ages ranging from 23 to 57 years, participated. One participant provided 100 samples, while the other participants each contributed 25. None of the subjects had vision problems. One subject participated in both data acquisitions (primary and secondary driver activities). All participants agreed to participate in this study and use the results for research purposes. In total, 2100 samples were collected for this investigation.

#### 3.1.1. Scenarios

The tests consisted of scenarios that serve as good representations of basic and distracting driving behaviors. Primary activity scenarios were chosen as recommended by the local Driving Exam Center (WORD) and were evaluated while the driving test was administered.

As stated in Tables No. 2 and No. 7, Appendix No. 2 of the Regulation of the Minister of Infrastructure of the Republic of Poland [63], these activities include:

- Passing through uncontrolled intersections (three- and four-way);
- Passing through intersections marked with signs establishing priority of passage;
- Drive through intersections with traffic lights;
- Drive through intersections where traffic flows around a traffic island;
- Perform one of the following parking maneuvers: perpendicular, angle, and parallel.

#### 3.1.2. Basic Driving Activities

**Figure 3:**
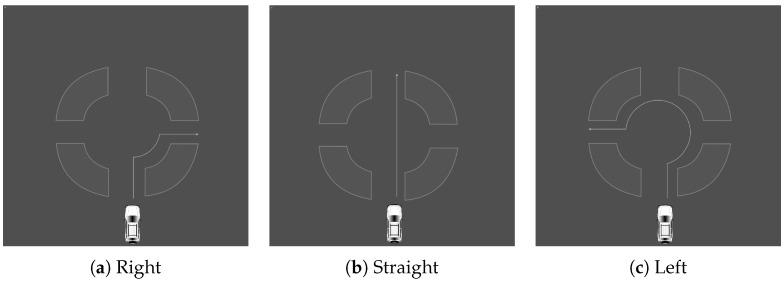
Roundabout scenarios such as ‘right’, ‘straight’, and ‘left’, (**a**–**c**), respectively.

**Figure 4:**
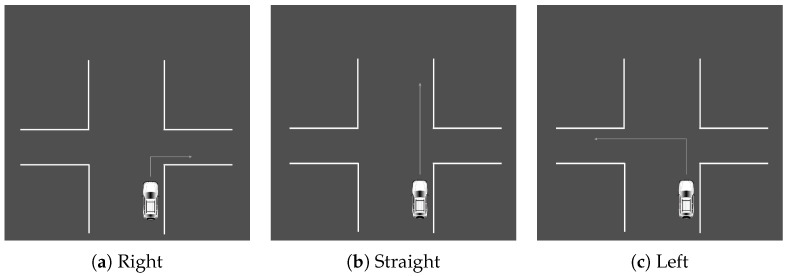
Crossroad scenarios such as ‘right’, ‘straight’, and ‘left’, (**a**–**c**), respectively.

**Figure 5:**
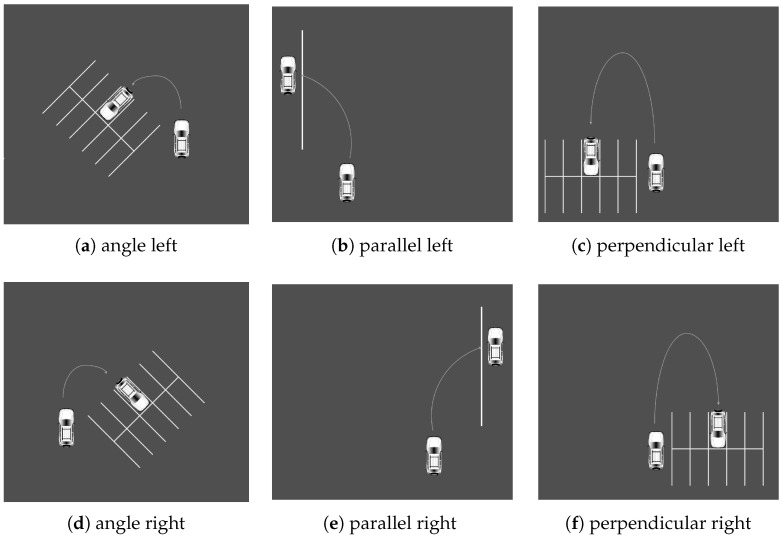
Parking scenarios such as ‘angle left’, ‘parallel left’, ‘perpendicular left’, ‘angle right’, ‘parallel right’, and ‘perpendicular right’, (**a**–**f**), respectively.

The driving simulator was used to carry out this experiment. To familiarize themselves with the machinery, each participant began with a test ride. Once they felt comfortable, a scenario was given and they were asked to complete the action while wearing JINS MEME ES_R Eyewear. To allow participants to concentrate solely on driving, the supervisor was in charge of managing the computer program and issuing voice commands. Three types of situation were created, each of which was performed in an appropriate setting. There were a total of 12 scenarios in this section. The first set of tasks was carried out in a roundabout. It involved making a left turn, a right turn, or going straight ahead, choosing the first, second, or third exit. The actions are illustrated in Figure 3. The second set of actions was executed at an intersection. The scenarios are similar to the roundabout. The second series of actions was carried out at a crossroad. The situations resemble those of a roundabout and are illustrated in Figure 4. The final set of situations comprises various parking methods, specifically, angle, parallel, and perpendicular parking. Each action was carried out twice, on each side of the street. All scenarios are illustrated in Figure 5.

#### 3.1.3. Distracting Driving Activities

The second investigation focused on secondary or distracting driving activities. They represent all actions that are performed when operating a vehicle that are not related to actual driving. However, they affect performance quality. These actions were carried out in a setting similar to sitting behind a wheel because they do not require being in a vehicle. This section of the study introduced four scenarios: eating, drinking, turning, and bending. Actions are explained in detail in Table 1.

#### 3.1.4. Data Format and Label Information

First, the data acquisition parameters are presented, followed by the header describing the content of each column that contains the sample number, the date in the format: dd.mm.rrrr:hh:mm:ss, and then the 3 channel accelerometer components:

- ACC${}_{X,Y,Z}:=$ acceleration on the X, Y, and Z axes.

Followed by the EOG sensor components:

- EOG${}_{L,R}:=$ raw EOG signal from the left and right eye, respectively;
- EOG${}_{H}:=$ the difference between the left and right eye potential (EOG${}_{L}$− EOG${}_{R}$);
- EOG${}_{V}:=$ negative arithmetical mean of the left and right eye potential − (EOG${}_{L}$ + EOG${}_{R}$)/2.

A list of dataframes comprising one sample signal is created by successively reading the data from the relevant path by folders. To accurately describe all signals, the rows containing the parameter specifications are removed, and the header is fixed. The labels for the primary activities are presented in Table 2.

### 3.2. Preprocessing

The data collected by smart glasses include signals from the four EOG channels (EOG${}_{L}$, EOG${}_{R}$, EOG${}_{H}$, and EOG${}_{V}$), three axes of the accelerometer (ACC${}_{X}$, ACC${}_{Y}$, and ACC${}_{Z}$) and three axes of the gyroscope (GYRO${}_{X}$, GYRO${}_{Y}$, and GYRO${}_{Z}$).

The signals collected by these sensors are often contaminated by noise and artifacts. For example, EOG channels can pick up electrical signals from the surrounding environment, which can cause baseline drift and power line noise. Linear and angular acceleration can be affected by vibrations or other disturbances, which can cause errors in measurements. To address these issues, various preprocessing techniques were applied to the data, which involves applying mathematical operations to the signals to remove unwanted components.

The first step of preprocessing was to apply a low-pass filter to remove power line noise (50 or 60 Hz, depending on the country) and baseline wandering. The next step was to use a band-pass filter to remove DC components of the EOG signal caused by electrode polarization.

After preprocessing the data, they can finally be analyzed using the statistical analysis and machine learning technique. Clean data provide valuable associations of changes in the EOG signal in the recognition of human behavior or cognition.

**Figure 6:**
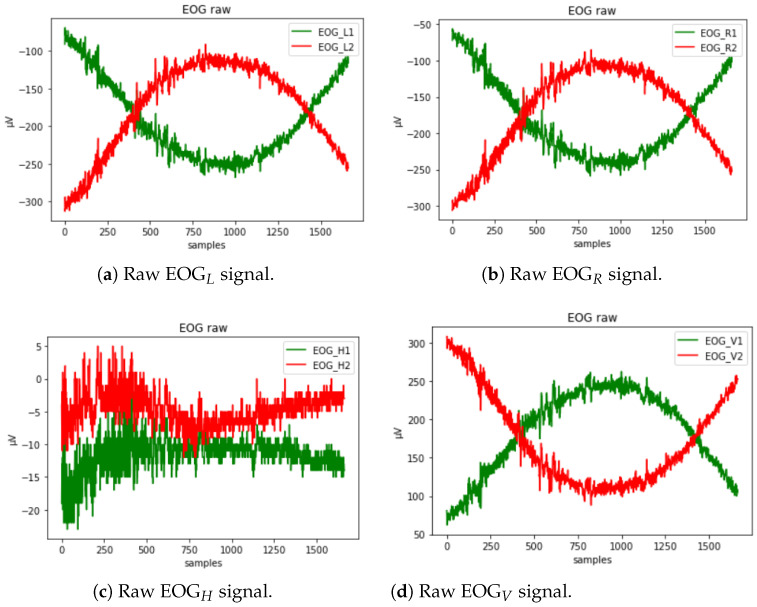
Raw EOG signal in channels EOG${}_{L}$, EOG${}_{R}$, EOG${}_{H}$, and EOG${}_{V}$ shown in (**a**–**d**), respectively.

The raw EOG signal presented in Figure 6 contains different types of artifacts that must first be filtered out. To reduce the noise from electricity lines and other potential types of noise, a second-order low-pass Butterworth filter is used to filter the EOG signal. It is applied to the signal twice: once forward and once backward. Such a filter has twice the order of the initial filter and zero phase. In addition, a slow unrelated alteration that is superimposed on the EOG signal, known as a baseline drift, might appear. It could be caused by a variety of things, including electrode polarization or interference with background signals [64]. To eliminate this effect, we have applied detrending by differentiating.

Linear acceleration signal in three axes undergoes preprocessing that consists of applying a median filter and a low-pass filter. The purpose of the median filter is to remove short irregular peaks. Since vigorous voluntary head rotations typically have frequencies below 20 Hz, a low-pass filter is applied to remove components with lower frequencies. However, this type of filter can introduce unwanted distortions while preserving low frequencies. To make the most of both techniques, they are combined by first applying the median filter and then passing the resulting signal through a low-pass filter with a Hamming window. A disadvantage of this approach is the potential weakening of values at the signal edges. However, these values were excluded due to the potential presence of noise caused by human control.

The entire dataset was then independently normalized using Z-score normalization. Z-score normalization helps distinguish the rest values and the values related to activities. The mean and standard deviation of each signal are calculated, and the samples values are replaced with the newly determined values using the following formula:

(1) $$x^{\prime }=\left(x-\mu \right)/\sigma ,$$

where μ is the mean of the signal, σ is the standard deviation, *x* is the current value of a sample, and *x* is the new value, so that the new mean of all values is 0 and the standard deviation is 1.

A sliding time window technique was used to segment all normalized sensor signals, with a window length of 5.6 s (280 samples) and a 50% stride (140 samples). Final samples were eliminated if the signal length was not divisible by 140.

**Figure 7:**
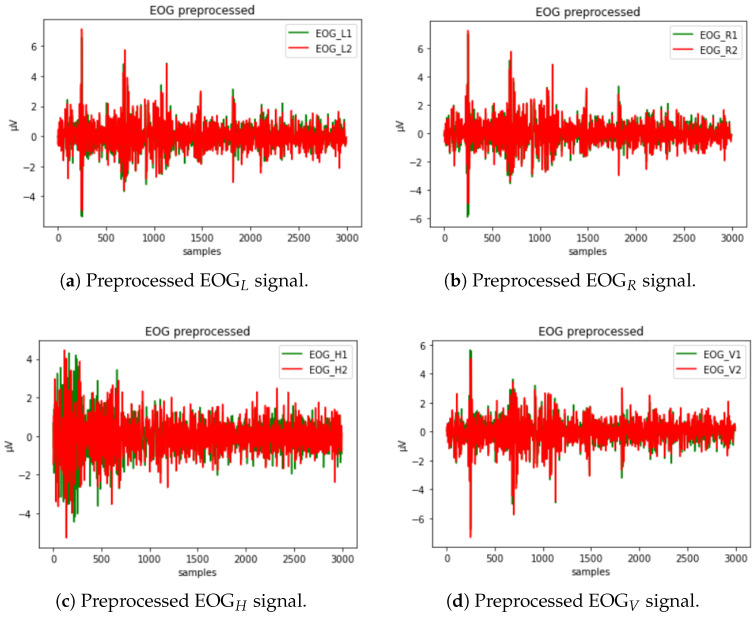
Preprocessed EOG signal in channels EOG${}_{L}$, EOG${}_{R}$, EOG${}_{H}$, and EOG${}_{V}$ shown in (**a**–**d**), respectively.

Completing some tasks required more time than for the others. Also, depending on the precision of the driver, the acquired signals had different lengths. The shortest one was obtained for secondary activity Turning back and lasted 103 samples (2.06 s); the longest one was for primary activity, while taking a left turn at a roundabout was 3013 samples (60.26 s) long. To train the model, the signal data were resampled at a rate of 3000. The results of signal preprocessing are shown in Figure 7.

### 3.3. Classification

In this study, we used 1D CNN for feature learning and classification. Multiple convolutional operators in CNN allow automatic recognition of important features from a higher-dimensional input [65,66,67,68]. Convolutions offer the advantage of taking into account the spatial organization of the data. In doing so, additional information about the position in relation to other samples is expected to be taken into account.

The 1D CNN can be used to analyze time series with one or more variables. The latter scenario involves combining two or more concurrent signals. On the basis of our previous experiments, we segmented the data using the sliding window segmentation technique (SWS). Different settings were tested to select the length *T* and stride size Δ*S* of a time window and the best values were chosen empirically. In 1D CNN, the only samples with an inherent ordering are those along the time dimension. The channels for the various variables do not have this, in contrast to the most popular 2D CNN.

**Figure 8:**
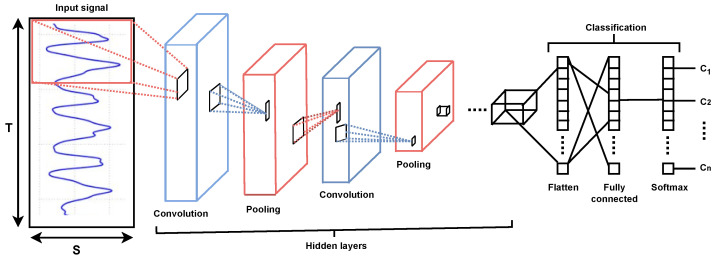
General architecture of a convolutional neural network for time series data classification. T represents time window, S represents the number of sensor channels, c represents a class, and n represents the number of classes.

The basic architecture of a CNN model is shown in Figure 8 and the parameters used in the 1D CNN are shown in Table 3. The first dimension of the input and output data is the batch size, the second dimension is the length of the sequence, and the third dimension is the number of features. The batch size was 32, the number of epochs was 100, and the learning rate (lr) was set to $2\times 10^{-4}$.

In terms of functionality, the model can be divided into two parts. The first component, common for this type of network, acts as a feature extractor. It matches templates using convolutional filtering techniques. To create the so-called “feature maps”, it uses layers and functions that include a convolutional layer, a batch normalization layer, a ReLU activation function, and a pooling layer. The network can learn higher-level features by being trained on a large dataset using a suitable number of epochs and a learning rate.

The second component is the categorization into one of the output classes. The input vector values are first reshaped using the global average pooling layer, a further dropout layer to prevent the model from overfitting, and a dense layer with the “softmax” activation function, which assigns the final label representing the predicted class value by performing a matrix vector multiplication. This process results in a new vector at the output.

### 3.4. Evaluation

The performance of the classifier was expressed in the form of tables with with the numbers of accuracy, precision, recall, and F1 score and confusion matrix.

Accuracy presents the percentage of correct predictions relative to all predictions made.

(2) $$\mathrm{Accuracy}=\frac{t_{p}+t_{n}}{t_{p}+t_{n}+f_{p}+f_{n}}\times 100\%,$$

where:

- True positive ($t_{p}$)—correctly classified trials;
- False positive ($f_{p}$)—incorrectly classified trials;
- True negative ($t_{n}$)—correctly classified nonevent trials;
- False negative ($f_{n}$)—incorrectly classified nonevent trials.

Precision is a metric that identifies the successful predictions of all predictions made in favor of the event.

(3) $$\mathrm{Precision}=\frac{t_{p}}{t_{p}+f_{p}}$$

Recall presents the fraction of correctly classified predictions of a particular activity with respect to all predictions made in favor of the activity.

(4) $$\mathrm{Recall}=\frac{t_{p}}{t_{p}+f_{n}}$$

The F1 score is a harmonic mean of precision and recall, which, compared to accuracy, should provide a more realistic model assessment in multiclass predictions with unbalanced classes.

(5) $$\mathrm{F1-score}=2\times \frac{\mathrm{Precision}\times \mathrm{Recall}}{\mathrm{Precision}+\mathrm{Recall}}$$

Categorical cross-entropy loss measures the model performance by comparing the actual and predicted labels according to the formula:

(6) $$\mathrm{CE}=-\sum_{i=1}^{N}t_{i}\times log\left(p_{i}\right),$$

where *t* is the true label, *p* the predicted label, and *N* the number of scalar values in the model output.

Linear acceleration and EOG signals that had already been analyzed were used to train and assess the network. A 9:1 ratio was used to divide the data into subsets for training and testing. A further division with 8:2 ratio was used on the training set to divide it into training and validation sets. Since the signals were sorted, the data had to be shuffled to train the model on signals from all possible classes.

## 4. Results

### Accuracy and Loss While Training

**Figure 9:**
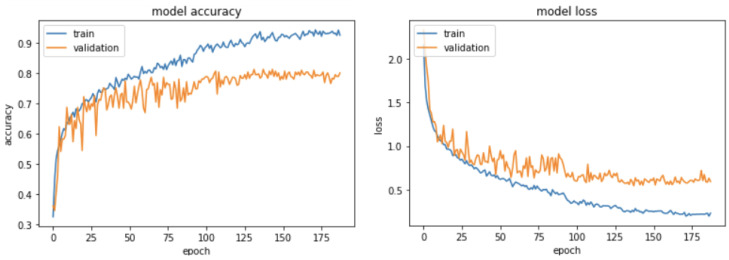
Accuracy and loss curve of the CNN model during the training and validation phases.

Figure 9 shows the accuracy curve for training and validation, and loss of the model with respect to the number of epochs elapsed. The loss function is categorical cross-entropy. When the epoch reached 130, the training accuracy was found to be greater than 90%, providing a loss value of 0.2. The validation rate was 80% with a loss of 0.6. The model obtained the optimal parameters in 188 epochs.

**Figure 10:**
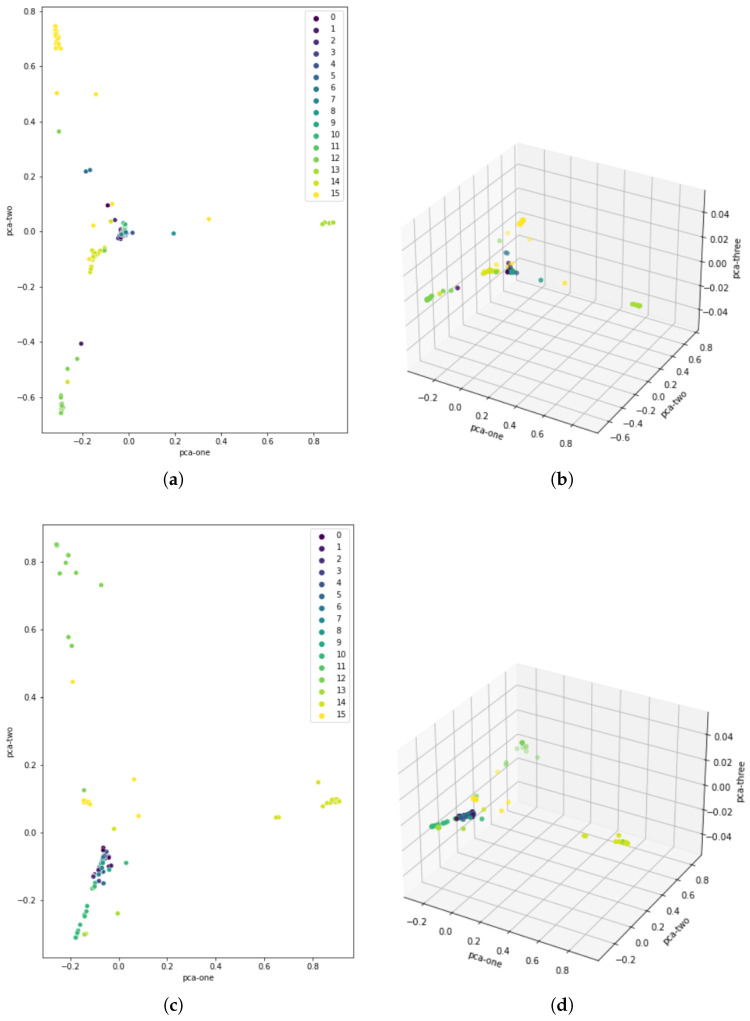
Scatter plot of the training and testing sets according to predicted labels: (**a**) 2D scatter plot of training set labels; (**b**) 3D scatter plot of training set labels; (**c**) 2D scatter plot of testing set labels; (**d**) 3D scatter plot of testing set labels.

Figure 10 shows how well the classes were segregated after 188 epochs in dimensions 2 and 3. A dimensionality reduction method known as principal component analysis (PCA) was used for visualization purposes. The correlation between different dimensions is used, and the goal is to provide as few variables as possible while preserving as much variation or information about the distribution of the original data as possible.

It can be seen that the distinction between primary and secondary driving activities is very apparent. The latter are also separated in such a way that they usually do not overlap. However, primary activities cover areas very close to each other, so the greatest misclassifications are anticipated.

The distinction between primary and secondary driving activities is readily apparent, as can be seen. The latter are divided in such a way that they do not primarily overlap. The greatest misclassifications were expected for groups of similar primary activities that covered areas that are relatively close to each other.

All activity predictions shown in Table 4 had a weighted average precision, recall, and F1 score of 0.83, 0.80, and 0.80, respectively. Drinking as a secondary activity is the category that has the best performance with all values equal to 1, while primary parallel parking on the left and perpendicular parking on the right are the categories that are mostly misclassified with F1 scores 0.42 and 0.44, respectively. In general, secondary driving actions performed better, all receiving F1 scores greater than 0.9.

**Figure 11:**
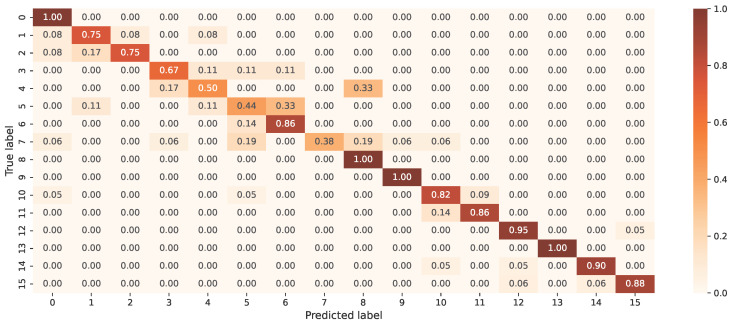
Confusion matrix of the driving activities classification.

The accuracy of the prediction of 15 driving actions is shown in Figure 11, where the accuracy of the prediction of each class is shown on the diagonal and inaccurate classifications are shown outside the diagonal.

Most misclassifications occurred in a group of activities that were related to each other. Parking activities show that the model had the most trouble detecting the difference between the same action being conducted on the left and right sides due to their similarity. The binary classification between secondary and primary activities has an accuracy rate of 99.5%. Although the latter was mistakenly classified as eating, the former was consistently assigned to the appropriate group.

An investigation of classifications for a particular collection of activities, including crossroad, parking, roundabout, and secondary activities, yielded accuracy ratings of 97.9%, 96.8%, 97.4%, and 99.5%, respectively. Table 5 displays these results together with the precision, recall, and F1 score values. Although the network received these actions as individual activities, it was still able to indicate patterns that differentiate the different types of action.

## 5. Discussion

Electrooculography (EOG) is a technique that is based on electrical features generated by the eye. By measuring the voltage difference between the cornea and the retina, it aims to capture the movements of the eyes [37]. JINS MEME ES_R Glasses (JINS Inc., Tokyo, Japan) are a smart glasses device that consists of a three-point electrooculography (EOG) and a six-axis inertial measurement unit (IMU) with a gyroscope and an accelerometer. They acquire ten channels: linear and angular acceleration on the X, Y, and Z axes, and four EOG channels: electric potentials on the electrodes on the right (EOG${}_{R}$) and left (EOG${}_{L}$), and the vertical (EOG${}_{V}$) and horizontal (EOG${}_{H}$) difference between them [61,69,70].

We have recognized road conditions based on electrooculograms acquired from drivers wearing JINS MEME ES_R smart glasses. The highest precision, recall, and F1 score for drinking (1.00 for each metric) were observed, whereas the lowest results were observed for parallel parking on the left side (precision of 0.44, recall of 0.4, and F1 score of 0.42).

Most misclassifications occurred in a group of activities that were related to each other, e.g., parking on the left and parking on the right side due to their similarity. The binary classification between secondary and primary activities has an accuracy rate of 99.5%. Although the latter was mistakenly classified as eating, the former was consistently assigned to the appropriate group.

In this study, the recognition of primary and secondary driver activities based on the processing of EOG signals with a convolutional network achieved excellent recognition performance, but there are still some limitations. The first limitation was to obtain the EOG signals in a simulated driving experiment. Although the experimental results showed that the turn or park condition was successfully induced and verified the effectiveness of the experimental scheme, it cannot be compared with the complexity of driving in real traffic. The second limitation was the limited number of experimental data segments. This setup could be used in future studies that do not expose volunteers to the dangers of real traffic. Classification models were trained on short signal samples. The third limitation was the use of only one time window width (5.6 s) to calculate the EOG characteristics without fully examining the impact of other time window divisions on the classification results.

## 6. Conclusions

In this paper, we introduced a CNN-based machine learning model to classify nine primary and four secondary car driver activities using physiological sensor data from JINS MEME ES_R (smart glasses):

1. We conducted a comparative analysis of wearable sensor data, including nine activities performed by the first driver and four activities performed by the second driver. Our proposed system achieves an impressive overall accuracy of 97% (±2) and an average F1 score of 95% (±2) in detecting these activities. Moreover, our model has the potential to prevent traffic accidents without requiring expensive safety equipment. To further validate our approach, future studies will involve acquiring additional data from real-world road conditions. Such an application would be beneficial for drivers, particularly older individuals or those with disabilities.
2. Research involves a comparative analysis of wearable sensor data obtained from different driving activities in various scenarios. By analyzing the signals collected from these sensors, researchers can assess the different activities performed by drivers, gaining insight into driver behavior and activity patterns in various driving scenarios. The developed system holds promise in preventing traffic accidents without the need for costly safety equipment.
3. Our investigation focuses on the utilization of sensor data from JINS MEME ES_R smart glasses and the development of an advanced machine learning model that can identify and classify primary and secondary activities of drivers. This state-of-the-art model learns the patterns associated with these activities and assigns them to their respective classes.
4. The use of JINS MEME ES_R sensor data involves analyzing and recognizing activities and scenarios associated with driving a vehicle. By integrating data from these wearable glasses, we created an efficient machine learning model that can learn activity patterns and accurately classify them into respective classes. This novel approach to the use of wearable sensor data offers valuable insights into driver behavior and activity recognition.
5. A notable contribution of this research is the realistic and noninvasive collection of data. The use of JINS MEME ES_R smart glasses provides a user-friendly and noninvasive method for gathering data during experiments. Unlike intrusive methods, these glasses capture data from the driver’s perspective without causing discomfort or interfering with the driving experience. This noninvasive approach ensures that the collected data closely resemble real-world driving scenarios, allowing for more precise analysis and classification of driver activities. By addressing the challenge of obtaining realistic data while prioritizing participant safety and comfort, this research underscores the importance of using such technology.

In summary, the main contributions of this research involve the utilization of JINS MEME ES_R sensor data, development of a machine learning model for activity recognition, comparative analysis of wearable sensor data, and a review of related approaches. These contributions improve understanding of driver behavior and activity recognition, potentially leading to improved driver safety and accident prevention when the time comes when autonomous car traffic with the participation of human drivers will become commonplace on the roads.

## Figures and Tables

**Table 1:** Secondary driving activities scenarios.

Eating	Drinking	Turning	Bending
Taking a bite of any food.	Taking a sip of water.	Turning back and reaching to a passenger’s seat.	Bending and picking up a fallen object.

**Table 2:** List of activities for which data were collected in this study (driving activities), using JINS MEME ES_R Smart Glasses.

Label	Activity
0	P_Crossroad_Left
1	P_Crossroad_Right
2	P_Crossroad_Straight
3	P_Parking_Diagonal_Left
4	P_Parking_Diagonal_Right
5	P_Parking_Parallel_Left
6	P_Parking_Parallel_Right
7	P_Parking_Perpendicular_Left
8	P_Parking_Perpendicular_Right
9	P_Roundabout_Left
10	P_Roundabout_Right
11	P_Roundabout_Straight
12	S_Bending
13	S_Drinking
14	S_Eating
15	S_Turning_Back

**Table 3:** CNN architecture with a fixed dropout rate of 0.4 and a minimum learning rate of $2\times 10^{-4}$.

Layer Name	No. Kernels (Units)	Kernel (Pool) Size	Stride Size	Activation
Convolution	128	5	1	ReLU
Batch norm	-	-	-	-
Max pooling	-	3	-	-
Convolution	128	5	1	ReLU
Batch norm	-	-	-	-
Convolution	128	5	1	ReLU
Batch norm	-	-	-	-
Global avg. pooling	-	-	-	-
Dense	2	-	-	Softmax

**Table 4:** Evaluation results (such as precision, recall, and F1 score) of driving activities.

Label	Activity	Precision	Recall	F1 Score
0	P_Crossroad_Left	1.00	0.67	0.80
1	P_Crossroad_Right	0.75	0.75	0.75
2	P_Crossroad_Straight	0.75	0.90	0.82
3	P_Parking_Diagonal_Left	0.67	0.75	0.71
4	P_Parking_Diagonal_Right	0.50	0.50	0.50
5	P_Parking_Parallel_Left	0.44	0.40	0.42
6	P_Parking_Parallel_Right	0.86	0.60	0.71
7	P_Parking_Perpendicular_Left	0.38	1.00	0.55
8	P_Parking_Perpendicular_Right	1.00	0.29	0.44
9	P_Roundabout_Left	1.00	0.88	0.93
10	P_Roundabout_Right	0.82	0.86	0.84
11	P_Roundabout_Straight	0.86	0.75	0.80
12	S_Bending	0.95	0.90	0.93
13	S_Drinking	1.00	1.00	1.00
14	S_Eating	0.90	0.95	0.93
15	S_Turning_Back	0.88	0.94	0.91

**Table 5:** Evaluation scores such as accuracy, precision, recall, and F1 scores, of the type-based classification.

Activity	Accuracy [%]	Precision	Recall	F1 Score
Crossroad	97.9	0.97	0.91	0.94
Parking	96.8	0.92	0.96	0.94
Roundabout	97.4	0.94	0.92	0.93
Secondary	99.5	0.99	1.00	0.99

## Data Availability

The data presented in this study are available on request from the corresponding author. The data are not publicly available due to privacy concerns.
